# Should I Stay or Should I Go? Seasonal Fluctuations of Wood Mouse Populations in Fields Surrounded by Woodlands

**DOI:** 10.3390/ani13122017

**Published:** 2023-06-17

**Authors:** Sara Savazza, Paola Bartolommei, Stefania Gasperini, Andrea Bonacchi, Emiliano Manzo, Roberto Cozzolino

**Affiliations:** Fondazione Ethoikos, Convento dell’Osservanza snc, Radicondoli, 53030 Siena, Italy

**Keywords:** rodents, *Apodemus*, Mediterranean, demography, fallow

## Abstract

**Simple Summary:**

The abundance and distribution of rodents are driving factors in shaping ecosystem structure and functioning. In the Mediterranean region, the wood mouse is among the main representatives of small mammal communities, inhabiting both fields and different woodland types. The former are strongly seasonal environments which can represent a temporarily suitable habitat for mice. We investigated the seasonal pattern of wood mouse occurrence in three different habitats (fields, oak forest, and coniferous plantation) in central Italy, monitoring the number of captures and the population structure of the wood mouse for three years. We found that, unlike woodlands, fields are less suitable in autumn–winter than in warmer months, being characterized in colder months by a lower number of mice, a lower number of adult and reproductive individuals, lighter individuals, and a higher number of resident mice. Conversely, in spring–summer, we observed an increase in individuals caught in fields, especially breeding adults. These seasonal variations provide evidence that fields can represent a suboptimal habitat in this area, whilst playing, at the same time, a potential role as a source of food and cover resources and mates for mice in spring–summer. Our study contributes in filling the knowledge gap on wood mice ecology in the Mediterranean region.

**Abstract:**

The wood mouse *Apodemus sylvaticus* is common in woodlands and open areas of the Western Palearctic. Despite extensive research, little is known about its population ecology in fields in the Mediterranean area, where the climate involves great seasonal changes in environmental features. Here, we investigated wood mice seasonal fluctuations in the number of captures and population structure by sampling long-fallow fields and woodlands, i.e., oak forest and conifer plantation, in a heterogeneous landscape of central Italy. Mice were live-trapped every two months for three years (23.814 trap-days). The number of captures, mice body weight, and proportion of adult, residents and breeding individuals were analyzed. Mice dynamics changed across seasons and habitats. In fields, we recorded more captures, more reproductive individuals, and fewer non-adults and resident individuals in the warmer months compared to the colder months; mice were heavier in warmer months. During the cold season, the captures and adult proportion in fields were lower than in resource-rich woodlands. Breeding and non-resident mice were more abundant in fields than in woodlands in warmer months. Overall, the seasonal demographic variations we recorded provide evidence that fields can represent a suboptimal habitat in Mediterranean heterogeneous landscapes, acting nonetheless as a source of food resources, cover, and mates for mice in spring–summer.

## 1. Introduction

The abundance and distribution of small mammals are driving factors in shaping ecosystem structure and functioning [1]. Rodents play a crucial role in supporting a wide range of predators, being a common prey for several species [2,3,4,5]. Moreover, they act as seed consumers and dispersers, directly affecting the natural regeneration and cover structure of plants [6,7], with cascading effects on all trophic levels. On the other hand, rodents also play an important role as reservoir hosts for vector-borne disease agents [8,9]. Thus, the knowledge of the occurrence and population dynamics of rodents is fundamental to understand the functioning of an ecosystem.

The wood mouse *Apodemus sylvaticus* is commonly found in the ground-dwelling rodent communities of the Western Palearctic. As a habitat generalist, this species can inhabit both open areas and different woodland types [10,11,12,13]. It is widespread in agro-forestry systems, when canopy and shrub cover are present. Although it is mainly granivorous, the wood mouse shows a marked feeding plasticity and it can also feed on fruits, plant green parts, fungi, and invertebrates [14,15]. It is, in turn, an important food source for many species of mammalian carnivores, raptors, and snakes [16,17,18]. It has been reported to show seasonal habitat preferences in agricultural landscapes, residing in woods and hedgerows during the winter months and moving to arable or unmanaged fields in the summer [19,20,21]. In this sense, a recent study conducted in a heterogeneous landscape of England has suggested that seasonal movements between patches may explain *A. sylvaticus* captures recorded in late spring, i.e., a decrease in abundance in woodlands and the associated increase in captures in arable lands [22]. Seasonal movements between habitats may be enhanced by density-dependent processes such as resource depletion, interference, or aggressive encounters, which push individuals, especially juveniles and subordinate ones, to leave and move to acquire resources or mates [23,24,25,26,27]. Seasonal fluctuations of mice populations can also be driven by the changes in vegetative cover and food availability, which are determined by the phenology of herbaceous species in fields and fallows. These strongly seasonal environments, when covered by dense and tall vegetation offering a high seed availability [19,22], can represent a temporarily suitable habitat for opportunistic species. However, population abundance alone is not able to define the role of habitat quality for the species (e.g., sink habitats inhabited by numerous individuals, [27,28,29]). Evaluating other population characteristics (e.g., age class, and reproductive and resident individuals) is thus needed to gain a deeper understanding of the habitat suitability and use by mice.

Seasonal variation in the abundance and population composition of *A. sylvaticus* has not been extensively studied in the Mediterranean area, where this species is among the main representatives of the ground-dwelling rodent communities and the climate involves great seasonal changes in environmental features. Taking into consideration these facts, we aimed to contribute in filling this knowledge gap studying the seasonal pattern of *A. sylvaticus* occurrence in a heterogeneous landscape of central Italy, characterized by long-fallow fields surrounded by woodlands. The latter are represented by recently coppiced oak forests and conifer plantations. Both have dense understory vegetation and high fruit production, as shown by the surveys we carried out in the same study area [30], and are likely to represent a higher quality habitat compared to open areas all year round. In fact, woodland trees and shrubs can provide vertical cover and refuges from predators (including avian and mammal ones) and additional food [29,30,31]. On the other hand, fields can provide trophic resources as well as protection from predators due to the herbaceous cover mainly from the end of spring to the beginning of autumn, according to the phenology of naturally occurring weedy species [32]. Based on the seasonal availability of food resources and vegetation refuges, we thus predicted that, unlike woodlands, fields would be less suitable in autumn–winter than in warmer months. In particular, compared to the latter period, fields in autumn–winter were expected to be characterized by a lower wood mice number, body mass, and overall number of resident and breeding individuals, whilst having a higher proportion of non-adult individuals. Furthermore, we expected to observe an opposite pattern in spring–summer, with an increase of individuals exploiting fields (especially breeding adults searching for mates), and a similar body mass of *A. sylvaticus* individuals across all habitat types.

## 2. Materials and Methods

### 2.1. Study Area

La Selva Forest (43°13′ N, 11°4′ E) is located 45 km from Siena, in central Italy, with an altitude between 350–700 m above sea level. It lies within the Mediterranean climatic region (sensu Köppen-Geiger classification, [33]) which is characterized by warm dry summers (mean monthly temperature of about 23 °C) and cool wet winters (mean monthly temperature of about 4 °C) with an average annual rainfall of about 750–1600 mm. The area appears as a mosaic of different habitat types including long-fallow fields surrounded by forests. The former had been cultivated up until ten years prior to the study, and, during the study, those fields were vegetated by spontaneous grasses and herbaceous plant species, without any anthropogenic activity. The surrounding lands are covered by deciduous woodland dominated by *Quercus cerris*, with a mixture of *Q. pubescens* and other deciduous woody species [34]. Coppiced stands were logged in different years and thus appear at different successional stages, with recent coppices being characterized by a high shrub cover and low tree density [30]. Furthermore, as unproductive lands were afforested with *Pinus nigra* and *P. halepensis* during the past century [35], the surrounding lands are also covered by conifer plantations with abundant shrub undergrowth [30]. *Apodemus flavicollis* and *Clethrionomys glareolus* (formerly *Myodes glareolus*), along with the wood mouse, constitute the ground-dwelling rodent community inhabiting both woodland types in our study area [30]. These two species are instead only occasionally recorded in fields within La Selva Forest [36].

### 2.2. Study Design

Three independent sampling areas (i.e., located at least 500 m apart [1]) were selected for each of the three habitat types (i.e., fields, oak forests, and conifer plantations). In each sampling area, we used a trapping grid with 49 traps (7 × 7 with traps spaced 10 m apart). To minimize the edge effect from neighboring habitats, the grids were placed at least 100 m apart from the habitat border. Each of the 9 grids was trapped every other month for three years, starting from September 2011 to July 2014, for a total of 18 trapping sessions. During each session, live traps (Sherman and LOT traps) were active for three consecutive nights, baited with a mixture of sunflower seeds, peanut butter, and apple, and provided with hemp nesting material. Trap check was conducted daily in the early morning. Captured individuals were identified at species level, sexed, aged, weighed, and reproductive status was assessed [37]. The weight of the animal was measured using *Pesola* spring balance (accuracy of 1 g). In accordance with [38], mice were assigned as juveniles, subadults, or adults when their weight was ≤13.0 g (juveniles), 13.5–19.5 g (subadults), or ≥20 g (adults). We cross-validated this weight-based classification through morphological traits such as moult and reproductive attributes and through information derived from recaptures of the same individuals. Evidence of reproductive activity was defined by visual parameters, such as development of testes for male, and development of nipples and opening of vagina for females. Pregnancy was defined according to abdomen form. Animals were marked by toe clipping and released at the place of capture. Although toe clipping is no longer recommended because of its possible implications for animal welfare [39], at the time of the study, this method was adopted by the scientific community for rodents also in field studies, as it ensures individual recognition throughout the animal’s life. Our study indeed is based on data collected only in the initial part of a larger investigation on rodent population dynamics where the loss of individual recognition would have undermined the validity of the entire research project. We have been using PIT tags for ten years now, as models of suitable size for our target species (1.25 × 8.5 mm) have become commercially available. Furthermore, since field identification of *A. sylvaticus* and its sister species *A. flavicollis* is particularly challenging in southern Europe, molecular analyses were performed in order to correctly identify the mice species [40]. DNA was extracted from ethanol-preserved ear clippings, which were dried under chemical hood for two hours and then digested overnight in 0.5 mL lysis buffer (100 mm Tris HCl pH 8.5; 5 mm EDTA; 0.2% SDS; 200 mm NaCl; 1 mg/mL Proteinase K) incubated at 37 °C. The molecular identification of species was performed through PCR with species-specific primers following the procedures and PCR conditions described in [41].

The field protocol for live trapping and manipulation of animals took place in compliance with the European Council Directive 92/43EEC (Italian law D.Lgs 157/92 and LR 3/1994) and with the European Council Directive 86/609/EEC (Italian law D.Lgs 116/92), and was approved by Regione Toscana, with the supervision of the committee of the Italian Institute for Environmental Protection and Research (ISPRA) (Regione Toscana, DR 5493/2010 and DR 6063/2012).

Finally, as vegetation height and cover are known to affect small mammal occurrence in fields [42,43], we measured grass height and cover in fields during trapping sessions in order to investigate their potential influence on number of mice captures. The mean grass height and the percentage of herbaceous cover (i.e., four percentage class: 0–25%; 25–50%; 50–75%; and 75–100%) were calculated by averaging four measurements inside two random quadrats (20 m × 20 m) per grid.

### 2.3. Statistical Analyses

Data of the three sampling areas were cumulated by habitat type. Data from different years were cumulated by trapping session. In order to assess potential seasonal changes in number of captures, composition of population, and individual body weight, trapping sessions were grouped by season, i.e., spring–summer (May, July, and September, hereafter referred to as “hot season”) and autumn–winter months (November, January, and March, hereafter referred to as “cold season”). We grouped sessions based on the availability of trophic and cover resources, which varies seasonally in our study area [31].

Differences in trappability are a general problem in field studies of small mammals, since they may lead to biased samples. We assumed mouse detectability to be constant over time and space, since it was found to not vary significantly among seasons in our study area during a previous investigation [30] and to not be affected by habitat type in similar study conditions [44]. However, we cannot exclude lower detectability of juveniles compared to adults due to trap-shy behavior [45].

The existence of significant differences in the number of captures among habitat types was explored for each session by Chi-squared test (χ^2^; [29,46]). The same test was used to compare the number of individuals caught both between hot and cold season and among the three habitat types, in accordance with [29]. The overall sex ratio was tested for deviations from the balanced sex ratio (1:1) in each habitat type through a two-sided binomial test, revealing no deviation from 1:1 in all three habitats (fields: *p* = 0.645; oak forest: *p* = 0.077; and conifer plantations: *p* = 0.439). Thus, merged data of both sexes were analyzed.

In order to estimate the proportion of reproductive individuals, we considered only potentially breeding mice (subadult and adult ones), whereas for the estimation of the age structure, we considered the proportion of non-adult individuals (juvenile and subadult ones). We excluded non-adult individuals and pregnant females from body weight analysis. Moreover, we computed the percentage of resident individuals, i.e., the proportion of mice captured more than once across sessions in the same trapping grid.

The number of breeding individuals, non-adult, and resident individuals in each habitat type were compared both between hot and cold season and among the three habitats by Chi-squared test (χ^2^; [29]). Mice weights were compared between hot and cold season by non-parametric Wilcoxon test and among the three habitat types using the non-parametric Kruskal–Wallis test [46]. If significant differences were found (*p* < 0.05), pair-wise comparisons were performed with a post-hoc Dunn’s test for multiple comparisons [47].

Correlation between grass height and number of captures in fields was evaluated by Kendall rank correlation coefficient [48].

All analyses were performed in R version 4.1.3 [49]. The package *dplyr* [50] and the package *ggplot2* [51] were, respectively, used for data manipulation and graphical outputs.

## 3. Results

We recorded 341 captures of 245 different wood mice (75 individuals in fields, 63 in oak forests, and 107 in conifer plantations) over 23,814 trap-nights. All rodents captured in fields belonged to *A. sylvaticus*, with the exception of two *A. flavicollis* and one *C. glareolus*. Conversely, in the oak forest, wood mice represented only 12% of overall captures (*A. flavicollis* 52% and *C. glareolus* 36%). Finally, conifer plantations were inhabited by all three species, with bank voles being captured less frequently (*A. flavicollis* 46%; *A. sylvaticus* 33%; and *C. glareolus* 21%).

**Figure 1:**
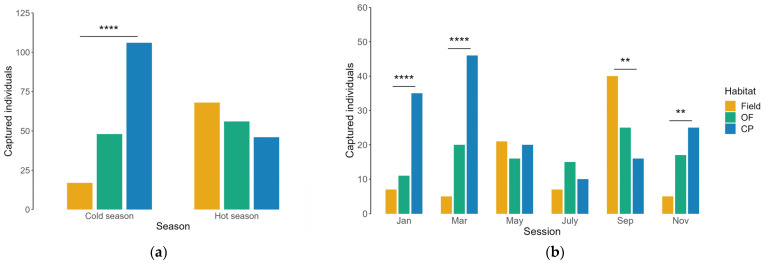
Number of wood mice caught in long-fallow fields (Field), recently coppiced oak forest (OF), and conifer plantations (CP) in central Italy over three years. Data were reported at: (**a**) seasonal scale (cold season: November, January, and March; hot season: May, July, and September); (**b**) capture session scale (every other month). Significant differences between treatments are indicated as ** *p* ≤ 0.01, **** *p* ≤ 0.001.

The number of *A. sylvaticus* captured was significantly different among habitat types in the cold season (χ^2^ = 71.614, df = 2, *p* < 0.001), whereas no differences were recorded during warmer months (χ^2^ = 4.282, df = 2, *p* = 0.118; Figure 1a). At a more detailed scale, the number of captures was significantly higher in conifer plantations in January (χ^2^ = 25.962, df = 2, *p* < 0.001), March (χ^2^ = 36.366, df = 2, *p* < 0.001), and November (χ^2^ = 12.936, df = 2, *p* = 0.002) (Figure 1b). In September, wood mice were more frequently captured in fields (49% of total captures) than in oak forests (20%) and conifer plantations (31%) (χ^2^ = 10.889, df = 2, *p* = 0.004), whereas in May (χ^2^ = 0.737, df = 2, *p* = 0.692) and in July (χ^2^ = 3.063, df = 2, *p* = 0.216), they were equally captured across all habitat types (Figure 1b). The number of captures was lower in the cold season than in the hot one in fields (χ^2^ = 30.6, df = 1, *p* < 0.001), while an opposite trend was found for conifer plantations (χ^2^ = 23.68, df = 1, *p* < 0.001; Figure 1a). The oak forest showed a comparable number of captures throughout the year (χ^2^ = 0.61, df = 1, *p* = 0.433; Figure 1a,b).

**Figure 2:**
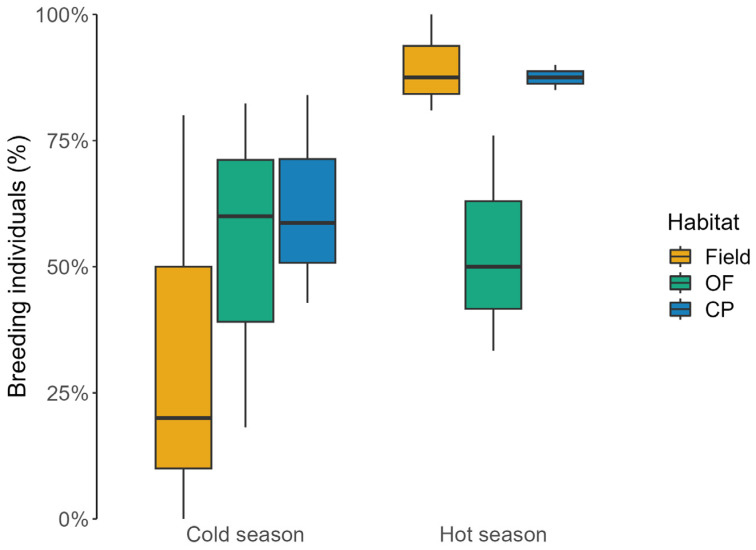
Percentage of breeding wood mice (median ± IQ) caught in long-fallow fields (Field), recently coppiced oak forest (OF), and conifer plantations (CP) in central Italy over three years. Data were reported at seasonal scale (cold season: November, January, and March; hot season: May, July, and September).

Breeding individuals made up most of the rodent captures, reaching 75% of overall captures (64 individuals) in fields, 68% (103 individuals) in oak forests, and 58% (60 individuals) in conifer plantations. The proportion of mice with evidence of reproductive activity differed across habitat types only during the hot season, but not in the cold one (hot season: χ^2^ = 19.414, df = 2, *p* < 0.001; cold season: χ^2^ = 4.425, df = 2, *p* = 0.109; Figure 2). Breeding individuals were less frequent in colder than in warmer months in fields and conifer plantations (fields: χ^2^ = 29.134, df = 1, *p* < 0.001; conifer plantations: χ^2^ = 6.831, df = 1, *p* < 0.01; Figure 2), whilst no seasonal differences were recorded in oak forests (χ^2^ = 0.062, df = 1, *p* = 0.803; Figure 2).

**Figure 3:**
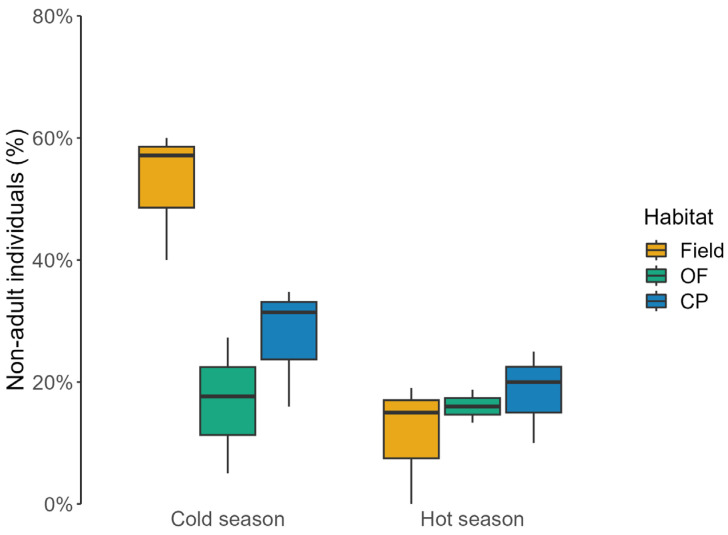
Percentage of non-adult wood mice (median ± IQ) caught in long-fallow fields (Fields), recently coppiced oak forest (OF), and conifer plantations (CP) in central Italy over three years. Data were reported at seasonal scale (cold season: November, January, and March; hot season: May, July, and September).

Adults comprised the bulk of the rodent populations, and the overall proportion of non-adult individuals did not significantly differ across habitat types (H = 1.93, df = 2, *p* = 0.38), reaching 22% of overall captures (19 individuals) in fields, 16% (16 individuals) in oak forests, and 28% (39 individuals) in conifer plantations. However, at the seasonal scale, the proportion of non-adult mice in fields was higher than in woodlands during the cold season, but not in the hot one (cold season: χ^2^ = 8.467, df = 2, *p* = 0.015; hot season: χ^2^ = 0.481, df = 2, *p* = 0.786; Figure 3). Non-adult mice were more frequent in the cold season than in the hot one in fields (χ^2^ = 11.455, df = 1, *p* < 0.001), whereas no seasonal differences were observed in woodlands (oak forest: χ^2^ = 0.007, df = 1, *p* = 0.934; conifer plantations: χ^2^ = 2.146, df = 1, *p* = 0.143; Figure 3).

**Figure 4:**
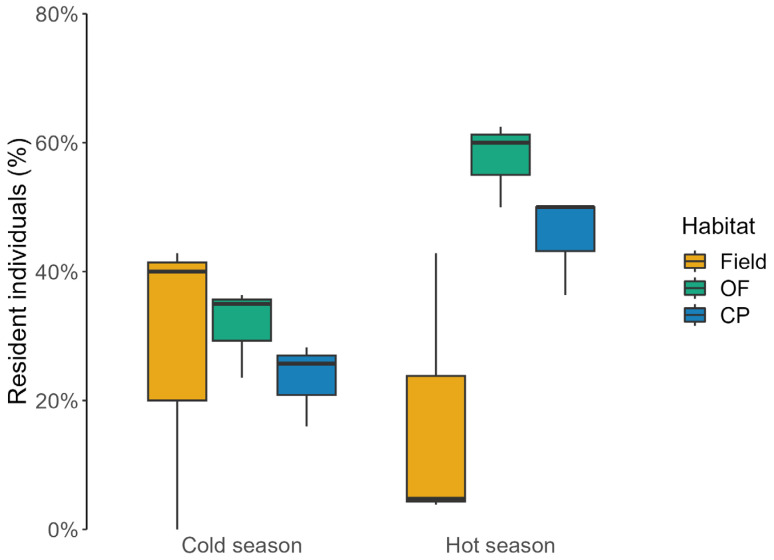
Percentage of resident wood mice (median ± IQ) caught in long-fallow fields (Field), recently coppiced oak forest (OF), and conifer plantations (CP) in central Italy over three years. Data were reported at seasonal scale (cold season: November, January, and March; hot season: May, July, and September).

In fields, the overall frequency of resident individuals was lower compared to woodlands, with 9% (seven individuals) of mice captured more than once in fields, 40% (25 individuals) in oak forests, and 31% (33 individuals) in conifer plantations. Resident individuals occurred with comparable frequency in all three habitat types in the cold season (χ^2^ = 0.819, df = 2, *p* = 0.664), whereas, in warmer months, we found inter-habitat differences in the percentage of residents (χ^2^ = 28.019, df = 2, *p* < 0.0001; Figure 4). Resident mice inhabited fields mainly in the cold season (χ^2^ = 4.339, df = 1, *p* = 0.037), whilst, in woodlands, the proportion of residents followed an opposite trend (oak forest: χ^2^ = 6.630, df = 1, *p* = 0.010; conifer plantations: χ^2^ = 6.622, df = 1, *p* = 0.010; Figure 4).

**Figure 5:**
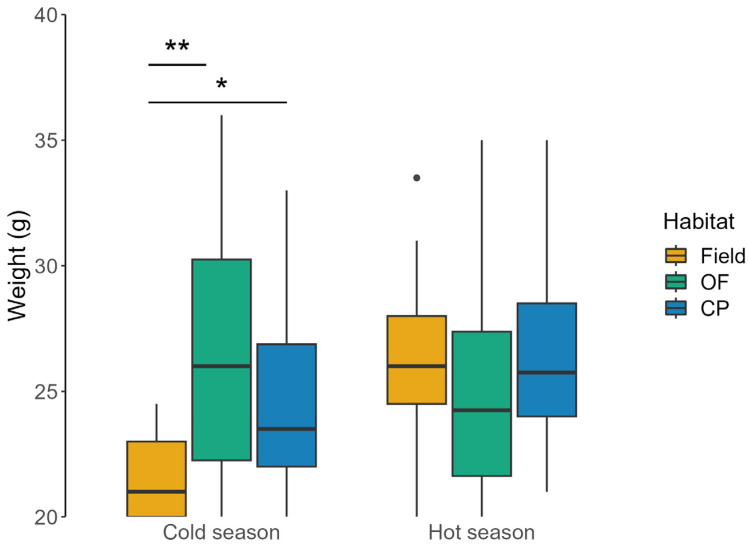
Body weight (median ± IQ) of wood mice caught in long-fallow fields (Field), recently coppiced oak forest (OF), and conifer plantations (CP) in central Italy over three years. Data were reported at seasonal scale (cold season: November, January, and March; hot season: May, July, and September). Significant differences between treatments are indicated as * *p* ≤ 0.05, ** *p* ≤ 0.01. Outliers are showed as •.

Body weight was significantly different across habitat types in the cold season (H = 9.6, df = 2, *p* = 0.008), with lower values in fields than in oak forests (*p* = 0.007; Figure 5) and in conifer plantations (*p* = 0.045; Figure 5). On the other hand, body weight did not significantly differ between the habitat types in the hot season (H = 5.45, df = 2, *p* = 0.066; Figure 5). The body weight of mice caught in fields was lower in the cold season than in the hot one (Z = 33.5, *p* < 0.001), whereas no difference was found for those captured in woodlands (oak forest: Z = 925, *p* = 0.052; conifer plantations: Z = 645, *p* = 0.068; Figure 5).

The percentage of herbaceous cover always fell within the highest class (i.e., 75–100%) in each grid in all trapping sessions, evidencing thus the presence of a continuous and persistent cover throughout the year. Grass height followed seasonal variations, varying from 20.4 (±10.4) cm in colder months to 58.2 (±23.4) cm in warmer months. However, we did not find a significant relationship between grass height and number of mice captures in our study area (Kendall’s τ = 0.153, z = 1.166, *p* = 0.244).

## 4. Discussion

The main aim of this study was to shed light on seasonal changes in the number of captures and population composition of wood mice in a seasonally suitable and usable habitat (i.e., fields) as opposed to surrounding woodlands (i.e., oak forests and conifer plantations) in a heterogeneous Mediterranean landscape. Our findings support the hypothesis that *A. sylvaticus* dynamics changed according to season and habitat type in our study area [22]. These results are supported by growing literature evidence suggesting that differences in habitat quality play an essential role in determining individual distribution, as well as in regulating temporal and spatial rodent population dynamics [29,52,53,54]. Similarly, seasons, in their turn, affect food availability and population density as a consequence of changes in environmental characteristics [19,29,55,56,57,58].

Wood mice occurrence varied seasonally in fields, where we recorded more captures, more adult and reproductive individuals, and fewer resident individuals in the hot season than in the cold one. As expected, the number of captures was lower in fields than in woodlands during the cold season. These results suggest that fields may better suit the ecological requirements of the species (i.e., food and cover resources) mainly in the warmer months, representing a seasonally suboptimal habitat for the wood mouse. In fact, in the fields of the study area, the main trophic resources (such as seeds of herbaceous species) are present from late spring to early autumn (Burrascano *pers. comm.*) and grass cover is the tallest from late spring to summer. Todd and colleagues [20] considered that, in farmlands, seasonal patterns in habitat use appear to be mostly a response to seasonal cover availability. In particular, in the winter, wood mice spent more time in hedgerows than in crop fields [20], as the sparse vegetation of the latter would expose rodents to a higher predation risk (e.g., by the tawny owl *Strix aluco*; [42]).

The number of captured mice and the percentage of breeding and resident individuals were found to vary seasonally also in conifer plantations. For this habitat, although the breeding trend is the same as in fields, the number of mice and the proportion of resident individuals follow instead an opposite trend. Conifer plantations differ from fields in the seasonal availability of trophic and cover resources. In this sense, the former are more similar to the recently coppiced oak forest in terms of fruit production and shrub cover, which are both available to mice all year round, as shown by the surveys we carried out in the same study area [30].

However, conifer plantations do not produce acorns [30], which are among the main trophic resources for mice found in oak forests [59,60], especially during cold months [14,59,60,61,62,63]. Nonetheless, we found wood mice to be more abundant in conifer plantations in the cold season than in the hot one. It is therefore likely that this habitat type is able to provide an adequate supply of trophic resources different from acorns, such as fruits and seeds, as already suggested by our former work [30] in the same study area. In fact, wood mice are characterized by a marked foraging plasticity that allows them to change their diet based on the most abundant available foods [63,64,65]. In a previous investigation in our study area, we observed that wood mice in autumn consumed different food items from several shrub species, in addition to acorns [30].

As for the oak forest, the wood mouse occurred with a comparable number of individuals and proportion of reproductive and non-adult individuals throughout the year. Recently coppiced oak forest actually represents an optimal habitat for the species in the study area, offering mice several food and cover resources available all year around [29,30,66].

The results on the body weight of adult individuals seem to confirm that seasonal variations in the number of captured individuals and population composition of wood mice are driven by seasonal resources availability [30,65]. In fact, according to our expectation, the body weight was found to be lower in the cold season than in the hot one in fields, but no significant variation was recorded in woodlands. Furthermore, mice were lighter in fields than in the woodlands during the cold season, but weighed similarly in the three habitat types in the warmer months. However, as *A. sylvaticus* is known to display strong density-dependent population regulation [67,68], we cannot exclude the idea that other factors (e.g., life history of surrounding populations and competition rates) may also have played a role in controlling the number of individuals and population structure in an unstable habitat such as the long-fallow fields.

Studies on the ecology of small mammals suggest that seasonal variations in habitat occurrence may also partially result from the movement of individuals between habitats [11,19,20,21,22]. Wood mice have been reported to change their habitat preference in different seasons, as they spend the winter in woodlands and hedgerows and move to arable fields in the summer as a result of the changes in resource availability throughout the year [19,20,21,22]. We did not trap mice in adjacent portions of the habitat to check for possible movements between habitats; thus, we do not have direct evidence of such phenomenon. Movements between habitats could nonetheless provide an explanation for our results: during colder months, wood mice could move away from the suboptimal habitat (i.e., fields) in search of food and mates, or driven by intraspecific competition. Indeed, during the cold season, fields are inhabited mostly by non-breeding and less competitive (i.e., non-adult) mice, as well as by lighter mice (i.e., individuals either subordinate or suffering from suboptimal habitat conditions; [27,29]). Conversely, in the hot season, the availability of resources in the fields may attract mice, which, however, would only pass through fields or reside there for a brief period of time and then move away in the autumn. In fact, during the warmer months, the population in fields is characterised not only by fewer resident individuals but also by a higher proportion of reproductive individuals, and the higher number of captures registered during that period suggests that the increase in mice captures might be primarily due to breeding animals looking for mates in fields. Mice movements between habitats could also be explained by the higher number of captures recorded in conifer plantations in the colder period, when the proportion of non-resident mice was higher than in the hot season, suggesting that the increase in the number of caught individuals we recorded could be attributed to roaming individuals. However, the hypothesis that mice move from one habitat to another contrasts with the lack of population growth during the cold season in oak forests, where the seasonal greater availability of acorns would be likely to attract mice.

At a more detailed scale, our data showed that mice captures in fields peaked in May and, especially, in September, whilst falling in July. The literature seems to confirm this result as wood mouse populations are known to tendentially decrease in full summer, due to the adverse climatic conditions of the Mediterranean region resulting in lower mice survival [30,56,58,69]. The adverse effect of both drought and high temperatures could be particularly sharp in fields, due to the structure and composition of the vegetation (e.g., lack of shrub and tree cover). However, despite the climatic adversity of hotter months, in our study area, reproduction occurred throughout the year and breeding individuals were always present in all three habitat types. This result is in contrast to the theory that the reproductive cycle of the wood mouse in the Mediterranean region occurs from autumn to spring, with a breeding pause in the summer [29], and suggests that climatic factors rather than reproduction might influence habitat use and occupancy and population parameters in our study area. As for fields, a supplementary analysis at the capture session scale shows that the proportion of non-adult individuals did not increase in September, suggesting that the mice population had not been mostly replaced. This likely implies that additional factors other than survival, such as variation in habitat use or reduced mice activity [20], could reflect the population decrease observed in fields in the harsh month of July. At the seasonal scale, the presence of breeding individuals throughout the year confirms that, in our study, the increased number of captures in the hot season cannot be explained by the recruitment of juvenile or subadult individuals into the adult population (differently from [70]). In fields, the number of non-adult individuals is indeed lower when captured mice are more abundant, whilst, in conifer plantations, the proportion of non-adult mice remains constant regardless of the number of caught individuals. 

Finally, although the grass height and cover are known to affect wood mouse occurrence in fields [25,43,44], in our study, the herbaceous cover did not act as a determinant of the number of mouse captures. However, other variables related to vegetation besides grass cover and height may be the driving factor in wood mouse fluctuations. Thus, it may be of interest to investigate the effect of food availability and quality (i.e., some vegetation offers nutrient-rich seeds and other mainly green biomass) as well as habitat structure and composition (e.g., presence of refuges and nesting sites) on field occupancy [55].

## 5. Conclusions

Our results confirm the existence of seasonal changes in wood mice occurrence in a heterogeneous landscape of the Mediterranean region. The recorded seasonal variations in the number of mice captures and population composition provide evidence that fields represent a suboptimal habitat in this area, whilst implying, at the same time, a potential role of these unwooded areas as a source of resources, mates, and space for this opportunistic species in the spring–summer. Further research is needed to understand the relative importance of life history (e.g., mortality and fecundity), intra- and inter-specific competition, and changes in food and cover availability in explaining seasonal variations in wood mouse occurrence.

## Data Availability

The data presented in this study are available as Supplemental Material.

## Supplementary Materials

The following supporting information can be downloaded at: https://www.mdpi.com/article/10.3390/ani13122017/s1, Supplementary Material S1: Mice-sampling data; Supplementary Material S2: Grass surveys.

## References

[1] Rosalino L.M., Ferreira D., Leitão I., Santos-Reis M. (2011). Usage patterns of Mediterranean agro-forest habitat components by wood mice *Apodemus sylvaticus*. Mamm. Biol..

[2] Macdonald D.W., Feber R.E., Tattersall F.H., Johnson P.J., Hutchings M., John E., Stewart A. (2000). Ecological experiments in farmland conservation. The Ecological Consequences of Environmental Heterogeneity.

[3] Mukherjee S., Goyal S.P., Johnsingh A.J.T., Pitman M.R.P.L. (2004). The importance of rodents in the Diet of jungle cat (*Felis chaus*), caracal (*Caracal caracal*) and golden jackal (*Canis aureus*) in Sariska Tiger Reserve, Rajasthan, India. J. Zool..

[4] Karell P., Ahola K., Karstinen T., Zolei A., Brommer J.E. (2009). Population dynamics in a cyclic environment: Consequences of cyclic food abundance on tawny owl reproduction and survival. J. Anim. Ecol..

[5] Pavey C.R., Nano C.E.M. (2013). Changes in richness and abundance of rodents and native predators in response to extreme rainfall in arid australia. Austral Ecol..

[6] Price M.V., Jenkins S.H., Murray D.R. (1986). Rodents as seeds consumers and dispersers. Seed Dispersal.

[7] Sone K., Hiroi S., Nagahama D. (2002). Hoarding of acorns by granivorous mice and its role in the population processes of *Pasania edulis* (Makino) Makino. Ecol. Res..

[8] Ferrari G., Girardi M., Cagnacci F., Devineau O., Tagliapietra V. (2022). First record of Hepatozoon spp. in alpine wild rodents: Implications and perspectives for transmission dynamics across the food web. Microorganisms.

[9] Dahmana H., Granjon L., Diagne C., Davoust B., Fenollar F., Mediannikov O. (2020). Rodents as hosts of pathogens and related zoonotic disease risk. Pathogens.

[10] Pollard E., Relton J., Hedges V. (1970). A study of small mammals in hedges and cultivated fields. J. Appl. Ecol..

[11] Ylonen H. (1991). Seasonal dynamics of small mammals in an isolated woodlot and its agricultiral surrondings. Ann. Zool. Fennici.

[12] Butet A., Paillat G., Delettre Y. (2006). Factors driving small rodents assemblages from field boundaries in agricultural landscapes of Western France. Landsc. Ecol..

[13] Wilson A.C., Fenton B., Malloch G., Boag B., Hubbard S., Begg G.S. (2014). Coexisting small mammals display contrasting strategies for tolerating instability in arable habitat. Eur. J. Wildl. Res..

[14] Abt K.F., Bock W.F. (1998). Seasonal variations of diet composition in farmland field mice *Apodemus* spp. and bank voles *Clethrionomys glareolus*. Acta Theriol..

[15] Bencini C., Bartolommei P., Bonacchi A., Cinque C., Gasperini S., Manzo E., Cozzolino R. Molecular insights into the dietary habits of ground-dwelling mediterranean rodents. Proceedings of the IV Convegno Italiano sui Piccoli Mammiferi.

[16] Bontzorlos V.A., Peris S.J., Vlachos C.G., Bakaloudis D.E. (2005). The diet of barn owl in the agricultural landscapes of Central Greece. Folia Zool..

[17] Carvalho J.C., Gomes P. (2004). Feeding resource partitioning among four sympatric carnivores in the Peneda-Gerês National Park (Portugal). J. Zool..

[18] Padial J.M., Âvila E., Sánchez J.M. (2002). Feeding habits and overlap among red fox (*Vulpes Vulpes*) and stone marten (*Martes Foina*) in two Mediterranean mountain habitats. Mamm. Biol..

[19] Ouin A., Paillat G., Butet A., Burel F. (2000). Spatial dynamics of wood mouse (*Apodemus sylvaticus*) in an agricultural landscape under intensive use in the Mont Saint Michel Bay (France). Agric. Ecosyst. Environ..

[20] Todd I.A., Tew T.E., Macdonald D.W. (2000). Arable habitat use by wood mice (*Apodemus sylvaticus*). 1. Macrohabitat. J. Zool..

[21] Tattersall F.H., Macdonald D.W., Hart B.J., Manley W.J., Feber R.E. (2001). Habitat use by wood mice (*Apodemus sylvaticus*) in a changeable arable landscape. J. Zool..

[22] Occhiuto F., Mohallal E., Gilfillan G.D., Lowe A., Reader T. (2021). Seasonal patterns in habitat use by the harvest mouse (*Micromys minutus*) and other small mammals. Mammalia.

[23] Schoener T.W. (1974). Resource partitioning in ecological communities. Science.

[24] Begon M., Townsend C.R., Harper J.L. (2006). Ecology: From Individuals to Ecosystems.

[25] Liu C., Sibly R.M., Grimm V., Thorbek P. (2013). Linking pesticide exposure and spatial dynamics: An individual-based model of wood mouse (*Apodemus sylvaticus*) populations in agricultural landscapes. Ecol. Modell..

[26] Malo A.F., Godsall B., Prebble C., Grange Z., McCandless S., Taylor A., Coulson T. (2013). Positive effects of an invasive shrub on aggregation and abundance of a native small rodent. Behav. Ecol..

[27] Martineau J., Pothier D., Fortin D. (2016). Processes driving short-term temporal dynamics of small mammal distribution in human-disturbed environments. Oecologia.

[28] Van Horne B. (1983). Density as a misleading indicator of habitat quality. J. Wildl. Manag..

[29] Navarro-Castilla Á., Barja I. (2019). Stressful living in lower-quality habitats? Body mass, feeding behavior and physiological stress levels in wild wood mouse populations. Integr. Zool..

[30] Gasperini S., Mortelliti A., Bartolommei P., Bonacchi A., Manzo E., Cozzolino R. (2016). Forest ecology and management effects of forest management on density and survival in three forest rodent species. For. Ecol. Manag..

[31] Gasperini S., Bonacchi A., Bartolommei P., Manzo E., Cozzolino R. (2018). Seasonal cravings: Plant food preferences of syntopic small mammals. Ethol. Ecol. Evol..

[32] Chang J., Ciais P., Viovy N., Soussana J.F., Klumpp K., Sultan B. (2017). Future productivity and phenology changes in European grasslands for different warming levels: Implications for grassland management and carbon balance. Carbon Balance Manag..

[33] Kottek M., Grieser J., Beck C., Rudolf B., Rubel F. (2006). World map of the Köppen-Geiger climate classification updated. Meteorol. Zeitschrift.

[34] Tognetti R., Cherubini P., Marchi S., Raschi A. (2007). Leaf traits and tree rings suggest different water-use and carbon assimilation strategies by two co-occurring *Quercus* species in a Mediterranean mixed-forest stand in Tuscany, Italy. Tree Physiol..

[35] Angiolini C., Landi M., Salerni E., Perini C., Piazzini S., Frignani F., Amici V., Geri F., Leonardi P., Pecoraro L. (2011). Piano di Gestione Naturalistico della Riserva Naturale di Cornocchia.

[36] Bonacchi A. (2014). Distribuzione di Micromammiferi Terricoli in Italia Centrale: Un Caso di Studio. Master’s Thesis.

[37] Gurnell J., Flowerdew J.R. (2006). Live Trapping Small Mammals: A Practical Guide.

[38] Navarro-Castilla Á., Barja I. (2014). Antipredatory response and food intake in wood mice (*Apodemus sylvaticus*) under simulated predation risk by resident and novel carnivorous predators. Ethology.

[39] Wever K.E., Geessink F.J., Brouwer M.A.E., Tillema A., Ritskes-Hoitinga M. (2017). A systematic review of discomfort due to toe or ear clipping in laboratory rodents. Lab. Anim..

[40] Bartolommei P., Sozio G., Bencini C., Cinque C., Gasperini S. (2015). Field identification of *Apodemus flavicollis* and *Apodemus sylvaticus*: A quantitative comparison of different biometric measurements. Mammalia.

[41] Michaux J.R., Kinet S., Filippucci M.G., Libois R., Besnard A. (2001). Catzeflis, F. Molecular identification of three sympatric species of wood mice (*Apodemus sylvaticus*, *A. flavicollis*, *A. alpicola*) in western Europe (Muridae: Rodentia). Mol. Ecol. Notes.

[42] Tew T.E., Macdonald D.W. (1993). The effects of harvest on arable wood mice *Apodemus sylvaticus*. Biol. Conserv..

[43] Benedek A.M., Sîrbu I. (2018). Responses of small mammal communities to environment and agriculture in a rural mosaic landscape. Mamm. Biol..

[44] Panzacchi M., Linnell J.D.C., Melis C., Odden M., Odden J., Gorini L., Andersen R. (2010). Effect of land-use on small mammal abundance and diversity in a forest—farmland mosaic landscape in South-Eastern Norway. For. Ecol. Manag..

[45] Rajska-Jurgiel E., Mazurkiewicz M. (2000). “Single-Capture” Rodents: Vagrants or just newly weaned young?. Acta Theriol..

[46] Sokal R.R., Rohlf J.R. (1997). Biometry: The Principles and Practice of Statistic in Biological Research.

[47] Zar J.H. (2010). Biostatistical Analysis.

[48] Green N.S., Wilkins K.T. (2014). Habitat associations of the rodent community in a grand prairie preserve. Southwest. Nat..

[49] R Core Team A Language and Environment for Statistical Computing. R: A Language and Environment for Statistical Computing.

[50] Wickham H., Averick M., Bryan J., Chang W., McGowan L.D., François R., Grolemund G., Hayes A., Henry L., Hester J. (2019). Welcome to the Tidyverse. J. Open Sourc. Softw..

[51] Wickham H. (2009). ggplot2: Elegant Graphics for Data Analysis.

[52] Pulliam H.R., Danielson B.J. (1991). Sources, sinks, and habitat selection: A landscape perspective on population dynamics. Am. Nat..

[53] Lin Y.T.K., Batzli G.O. (2001). The influence of habitat quality on dispersal, demography, and population dynamics of voles. Ecol. Monogr..

[54] Janova E., Heroldova M., Konecny A., Bryja J. (2011). Traditional and diversified crops in south Moravia (czech republic): Habitat preferences of common vole and mice species. Mamm. Biol..

[55] Fernandez F.A.S., Dunstone N., Evans P.R. (1996). Population dynamics of the wood mouse *Apodemus sylvaticus* (Rodentia: Muridae) in a Sitka spruce successional mosaic. J. Zool..

[56] Díaz M., Torre I., Arrizabalaga A. (2010). Relative Roles of Density and Rainfall on the Short-Term Regulation of Mediterranean Wood Mouse Apodemus Sylvaticus Populations. Acta Theriol..

[57] Janova E., Heroldova M. (2016). Response of small mammals to variable agricultural landscapes in Central Europe. Mamm. Biol..

[58] Sunyer P., Muñoz A., Mazerolle M.J., Bonal R., Espelta J.M. (2016). Wood mouse population dynamics: Interplay among seed abundance seasonality, shrub cover and wild boar interference. Mamm. Biol..

[59] Mortelliti A., Sozio G. (2015). Empirical evaluation of the strength of interspecific competition in shaping small mammal communities in fragmented landscapes. J. Geotech. Geoenviron. Eng. ASCE.

[60] Bonacchi A., Bartolommei P., Gasperini S., Manzo E., Cozzolino R. (2017). Acorn choice by small mammals in a Mediterranean deciduous oak forest. Ethol. Ecol. Evol..

[61] Hansson L. (1985). The food of bank voles, wood mice and yellow-necked mice. Symp Zool Soc Lond..

[62] Corbet G.B., Harris S. (1981). The Handbook of British Mammals.

[63] Khammes N., Aulagnier S. (2007). Diet of the wood mouse *Apodemus sylvaticus* in three biotopes of Kabylie of Djurdjura (Algeria). Folia Zool..

[64] Sunyer P., Espelta J.M., Bonal R., Muñoz A. (2014). Seeding phenology influences wood mouse seed choices: The overlooked role of timing in the foraging decisions by seed-dispersing rodents. Behav. Ecol. Sociobiol..

[65] Unnsteinsdottir E.R., Hersteinsson P. (2011). Effects of Contrasting Habitats on Population Parameters and Diet of Apodemus Sylvaticus (Rodentia) in South-Western Iceland. Mammalia.

[66] Alcántara M., Díaz M. (1996). Patterns of body weight, body size, and body condition in the wood mouse *Apodemus sylvaticus* L.: Effects of sex and habitat quality. Proceedings of the 1st European Congress of Mammology.

[67] Montgomery W.I. (1989). Population regulation in the wood mouse, *Apodemus sylvaticus*. I. Density-dependence in the annual cycle of abundance. J. Anim. Ecol..

[68] Mallorie H., Flowerdew J.R. (1994). Woodland small mammal population ecology in Britain. A preliminary review of the Mammal Society survey of wood mice (*Apodemus sylvaticus*) and bank voles (*Clethrionomys glareolus*). Mammal. Rev..

[69] Torre I., Arrizabalaga A., Díaz M. (2002). Ratón de campo (*Apodemus sylvaticus* Linnaeus, 1758). Galemys.

[70] Canova L., Maistrello L., Emiliani D. (1994). Comparative ecology of the wood mouse *Apodemus sylvaticus* in two differing habitats. Z. Saugetierkd.

